# Use of 13-valent pneumococcal conjugate vaccine in children older than 5 years of age

**DOI:** 10.1186/1824-7288-40-S1-A1

**Published:** 2014-08-11

**Authors:** Nicola Principi, Susanna Esposito

**Affiliations:** 1 Pediatric Department of Pathophysiology and Transplantation, Università degli Studi di Milano, Fondazione IRCCS Ca’ Granda Ospedale Maggiore Policlinico, Milan, Italy

## 

The first pneumococcal conjugate vaccine (PCV7) included 7 serotypes and was licensed for the use in infants and in children < 5 years. Recently, preparations containing a greater number of pneumococcal serotypes have been developed and one of them, that including the greatest number of serotypes (PCV13), has been licensed for the use in older children and adolescents. Compared to young children and the elderly, children and adolescents aged 6-17 are at lower risk of pneumococcal disease. Disease incidence falls markedly after the second year and reaches its lowest point in adolescence. Moreover, also carriage of *Streptococcus pneumoniae* (Sp) also declines as children reaches adolescence. Consequently, it is thought that vaccination of older children and adolescents could only marginally reduce the number of Sp disease in these subjects and poorly contribute toward herd immunity for unvaccinated individuals. However, some recent data regarding carrier state of healthy older children and adolescents vaccinated with PCV7 during infancy and living in areas with vaccination coverage lower than 70% seem to suggest that, at least in some particular situations, Sp carriage can be higher than expected and regard most of the serotypes included in the vaccine. This could indicate that mucosal protection offered by PCV7 wanes in time and that after some years, vaccinated children when exposed to serotypes included in the vaccine can be re-colonized with these serotypes and become potential source of infection for unvaccinated subjects. Vaccination with PCV13 could be useful to reduce this risk. However, the most striking evidence of the potential beneficial effect of vaccination with PCV13 of older children and adolescents regards the so called subjects at risk. There is a not marginal number of subjects 6-17 years old with chronic heart, kidney, liver and respiratory disease, diabetes, cochlear implants or cerebrospinal fluid leaks, functional or anatomic asplenia or immunosuppression for whom an increased risk of severe pneumococcal disease is demonstrated or rationally supposed. In these subjects, administration of PCV13 is today strongly recommended, with different schedules according to the previous use of the 23-valent polysaccharide vaccine (PP23). On the other hand, PP23, since long time suggested for protection of subjects at risk >2 years of age, has several problems including an absolute poorer immune response to common serotypes in comparison to PCV13, an hyporesponsiveness to repeated doses, and an uncertain efficacy in risk groups.

